# Global epidemiology of serogroup Y invasive meningococcal disease: a literature review

**DOI:** 10.1017/S0950268824001535

**Published:** 2024-12-05

**Authors:** Myint Tin Tin Htar, Jamie Findlow, Paul Balmer, David Swerdlow

**Affiliations:** 1 Vaccine Medical Development and Scientific/Clinical Affairs, Pfizer Inc, Paris, France; 2 Vaccine Medical Development and Scientific/Clinical Affairs, Pfizer Ltd, Tadworth, UK; 3 Vaccine Medical Development and Scientific/Clinical Affairs, Pfizer Inc, Collegeville, PA, USA

**Keywords:** bacteria, clinical manifestiations, infectious disease, meningococcal, serogroups, vaccine

## Abstract

Serogroup epidemiology of invasive meningococcal disease (IMD) is constantly evolving, varying by time and location. Surveillance reports have indicated a rise in meningococcal serogroup Y (MenY) in some regions in recent years. This systematic literature review explores the evolving epidemiology of MenY IMD globally based on review of recent articles and national surveillance reports published between 1 January 2010 and 25 March 2021. Generally, MenY incidence was low (<0.2/100,000) across all ages in most countries. The reported incidence was more frequent among infants, adolescents, and those aged ≥65 years. More than 10% of all IMD cases were MenY in some locations and time periods. Implementation of vaccination evolved over time as the rise in MenY IMD percentage occurred. Cases decreased in countries with quadrivalent vaccine programs (e.g., United Kingdom, the Netherlands, United States, and Australia), whereas the MenY burden increased and made up a large proportion of cases in areas without vaccine programs. Continuous monitoring of epidemiologic changes of IMD is essential to establish MenY burden and for implementation of prevention strategies.

## Introduction

Invasive meningococcal disease (IMD), caused by the bacterium *Neisseria meningitidis*, is a major global health concern associated with high rates of morbidity and mortality [1]. The disease is fatal in up to 50% of untreated cases, and survivors can be left with debilitating long-term sequelae, including hearing loss, cognitive difficulties, and visual disturbances [1, 2]. The global burden of IMD is substantial, with approximately 1.2 million cases of disease occurring each year and resulting in approximately 135,000 deaths worldwide [3]. Infants and, to a lesser extent, children <5 years of age, have the highest risk for disease, and a second, smaller peak in adolescents and young adults is observed in some geographic regions [2, 4–6].

Although there are 12 serogroups of *N meningitidis*, only 6 (A, B, C, W, X, and Y) have caused epidemics and are associated with serious invasive disease [2], with serogroups ABCWY generally causing most IMD [7, 8]. Serogroup X is rare but has been associated with localized outbreaks in Africa [5]. Over the last few decades, many countries have implemented meningococcal vaccines into their routine immunization programs [7, 8]. With the recent increase in meningococcal serogroup W, a shift has occurred from monovalent meningococcal serogroup C (MenC) vaccines towards quadrivalent serogroups A, C, W, and Y (MenACWY) conjugate vaccines in the national immunization programs to provide broader protection against IMD [9].

The serogroup epidemiology of IMD is constantly evolving and can vary over time and geographic location [2]. The different serogroups are more prevalent in various regions over time [4]. For instance, surveillance reports have identified a rise in meningococcal serogroup Y (MenY) throughout Europe and Australia in recent years [10]. Understanding changes in serogroup-specific incidence and the relative distribution of different serogroups among all IMD is important when considering meningococcal immunization policies [11]. To our knowledge, there have been no recent articles on the global burden of MenY disease. This literature review describes MenY IMD incidence and the percentage of IMD cases caused by MenY worldwide from the literature published from 1 January 2010 to 25 March 2021.

## Methods

**Figure 1.**
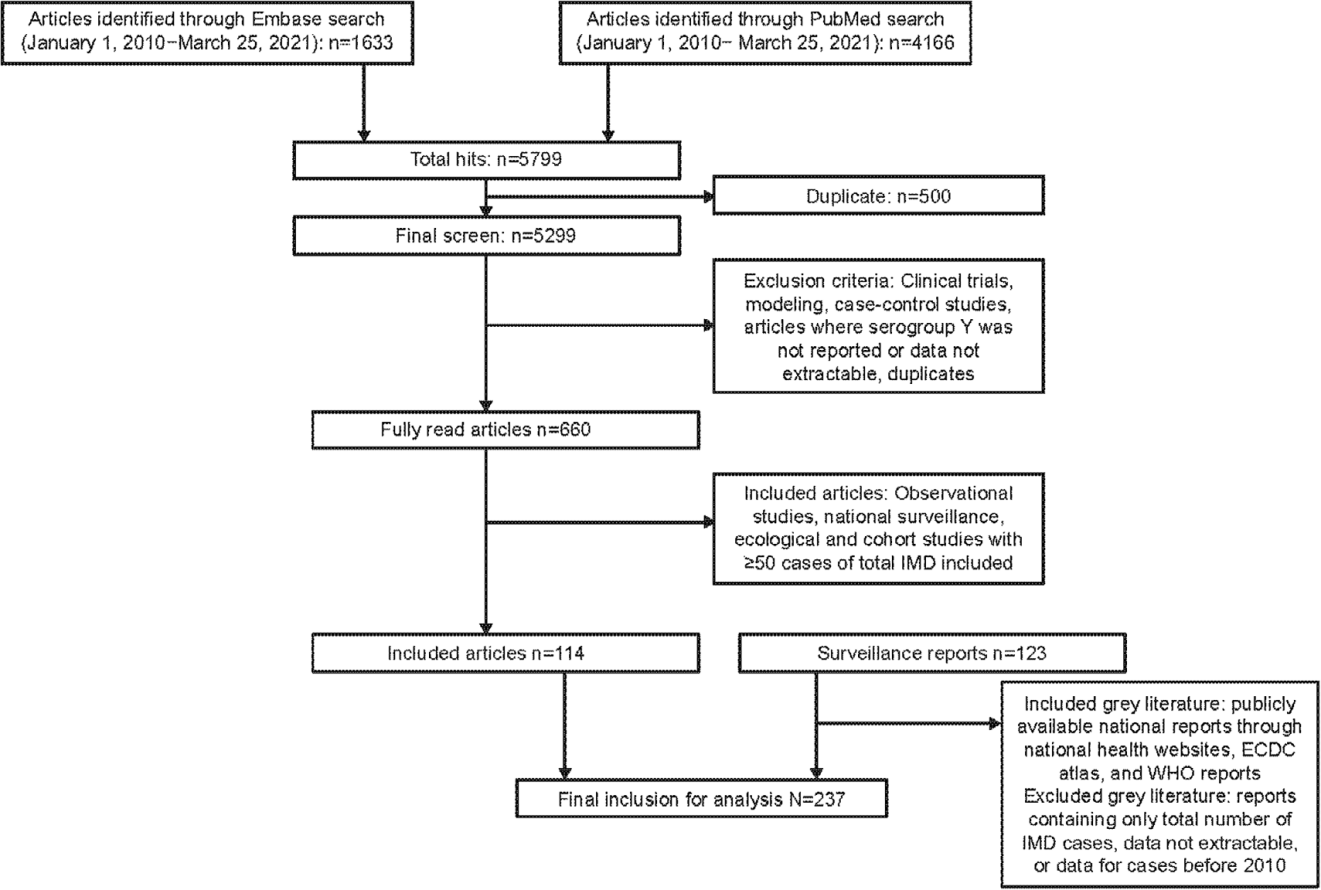
Flowchart of included literature. ECDC, European Centre for Disease Prevention and Control; IMD, invasive meningococcal disease; WHO, World Health Organization.

We conducted a literature search of MEDLINE (through PubMed) and Embase publications and national surveillance system reports (grey literature) containing IMD epidemiology data. The search was restricted to publications and reports published between 1 January 2010 and 25 March 2021. The grey literature search was performed through websites previously known to the authors to report subnational, national, and multinational IMD epidemiology, including European Centre for Disease Prevention and Control (ECDC) atlas, and World Health Organization reports (Figure 1). The documents were searched for ‘meningococcal’ and ‘meningitidis’ to determine relevant IMD data. Refer to the Supplementary Material for the full search string. Data from observational studies, national surveillance studies, and ecological and cohort studies with ≥50 cases of total IMD were included. Publications and grey literature reports containing data of only the total number of IMD cases or reporting data only before 2010 were excluded. Reports and publications in which MenY was not reported or data were unable to be extracted were also excluded. In cases of duplicate data, the grey literature sources were considered primary, with later reports and reports from larger geographic areas taking precedence. Publications identified through PubMed and Embase searches were evaluated per the inclusion and exclusion criteria by two authors. Single-centred studies, specific outbreak reports, meta-analyses, systematic reviews, clinical trials, case reports, modelling studies, randomized trials, nonhuman studies, and case–control studies were excluded. Data on the incidence of MenY disease overall and by age groups and the percentage of MenY disease among IMD overall and by age groups were extracted. Serogroup Y data were collected regardless of diagnostic or confirmatory methods, or the case definition used. Incidence data were extracted per publications or reports (no additional calculations made), and the percentage of serogroup Y among all IMD was taken either as available in the references or calculated by the number of serogroup Y divided by the number of total IMD evaluated for serogroups. Data on incidence or percentage of different clinical manifestations among all serogroup Y were collected, if available. Analyses of incidence and proportion of serogroup Y among all IMD were descriptive and performed using Microsoft Excel. To place data in context, extracted incidence results across years were considered alongside available country-specific data regarding vaccination programs.

## Results

A total of 5,799 articles were identified, and after excluding duplicates, 5,299 were screened. We reviewed 660 articles in full, and 114 articles and 123 reports met the inclusion criteria (Figure 1). Data are included from more than 80 countries spanning 6 continents. The covered data periods included 1997–2019.

**Figure 2.**
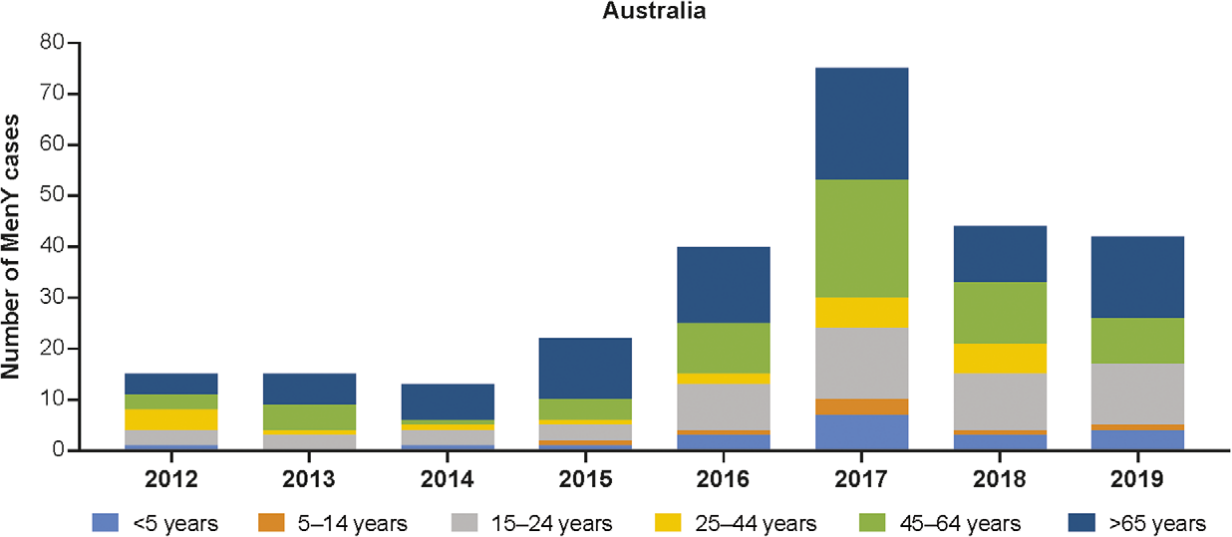
Number of MenY IMD cases in Australia by age group [12]. IMD, invasive meningococcal disease; MenY, meningococcal serogroup Y.

**Figure 3.**
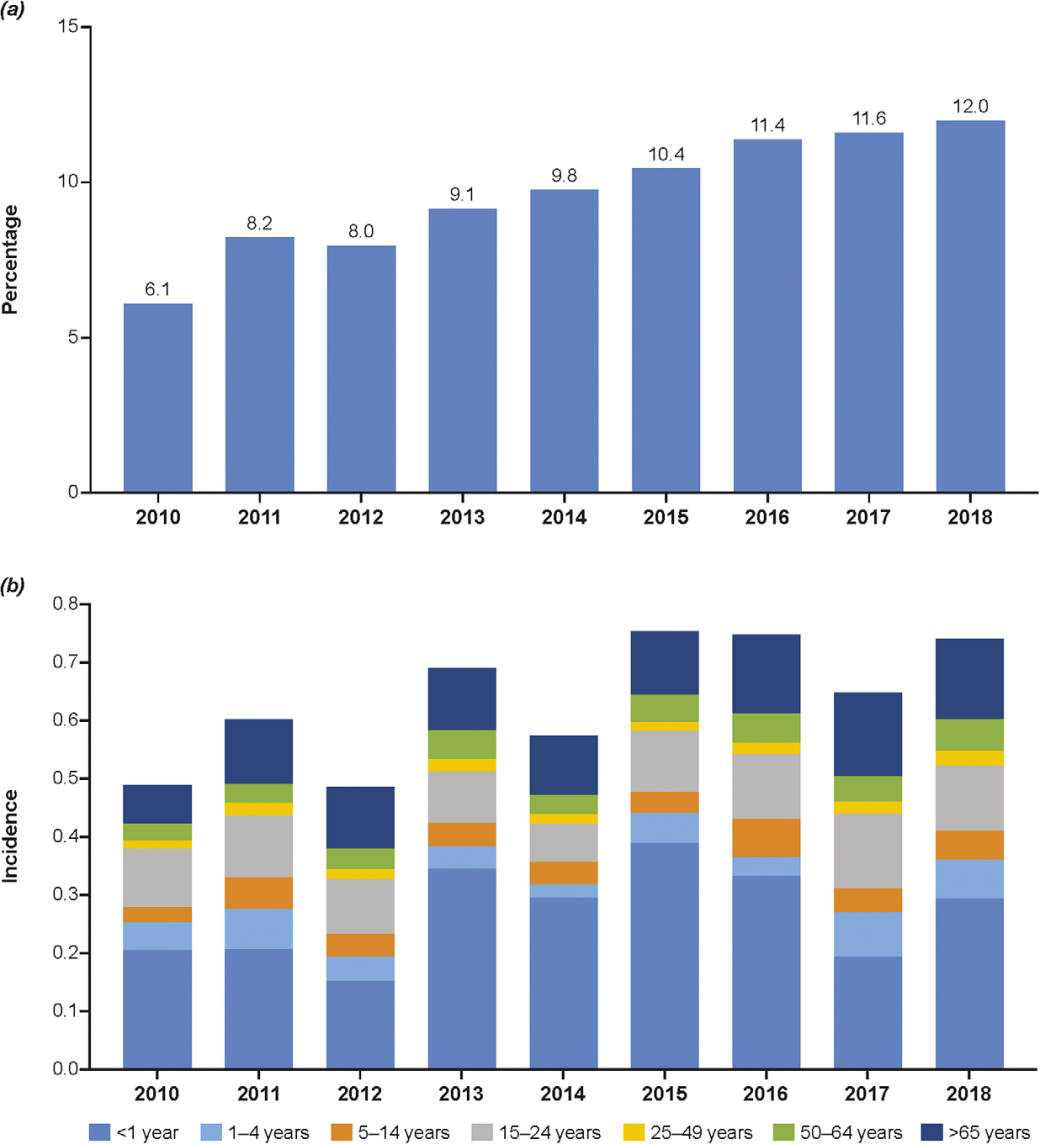
Percentage of IMD cases due to MenY overall (a) and incidence of MenY by age group in the European Union/European Economic Area (b) [10]. MenY, meningococcal serogroup Y.

Among the six serogroups associated with IMD previously mentioned, reports indicate a rise in the incidence of MenY IMD in multiple global regions (Figures 2 and 3). Trends in MenY incidence rates in selected countries are shown in Table 1. One key trend observed was the decreasing rate of MenY in countries with an active vaccination program against this serogroup [6, 10]. Although not analysed for serogroup Y individually, the benefit of MenACWY vaccination in the United States has presumably contributed to the 20%–95% decrease in the incidence of serogroups A, C, W, and Y between 2008 and 2015 for individuals <20 years of age [6, 22]. Revisions to immunization programs have recently been made in the United Kingdom, Spain, the Netherlands, and Australia [23]. In these countries, the MenACWY vaccine replaced the MenC-only vaccine. The United Kingdom has seen a decrease in MenY IMD since the vaccine introduction in 2015 [23], with cases dropping from 120 in 2015 to 81 in 2018 across all age groups (Supplementary Figure S1) [10]. In the Netherlands, the number of MenY cases dropped from 24 in 2018 to 17 in 2019 after the vaccine was implemented [10, 23].

**Table 1.** Incidence of MenY disease across all age groups

Publication	Country	Time period	Incidence MenY (per 100,000)
*Africa*			
du Plessis et al. [13]	South Africa	2005	0.11
		2008	0.04
Soeters et al. [14]	Sub-Saharan (five countries)	2015–2017	0.01
*Australia*			
Archer et al. [15]	Australia	1999	0.03
		2000	0.07
		2001	0.04
		2002	0.07
		2003	0.05
		2004	0.04
		2005	0.05
		2006	0.02
		2007	0.06
		2008	0.04
		2009	0.04
		2010	0.04
		2011	0.05
		2012	0.07
		2013	0.06
		2014	0.05
		2015	0.09
*Europe*			
ECDC	EU/EEA (31)	2010	0.04
		2011	0.06
		2012	0.05
		2013	0.06
		2014	0.05
		2015	0.06
		2016	0.07
		2017	0.07
		2018	0.07
*Latin America*			
Tauil et al. [16]	Brazil	2005–2009	0.03
		2010	0.04
		2011	0.04
*North America*			
Cohn et al. [17]	United States	1998–2007	0.18
MacNeil et al. [6]	United States	2006–2015	0.06
ABC Report^a^ [18]	United States	2010	0.07
		2011	0.07
		2012	0.05
		2013	0.02
		2014	0.01
		2015	0.02
		2016	0.02
		2017	0.02
		2018	0.03
EMDS report [19]	United States	2015	0.01
		2016	0.02
		2017	0.01
		2018	0.01
Brown et al. [20]	Canada (Ontario)	2000–2006	0.18
Li et al. [21]	Canada	2006–2011	0.10
		2006	0.08
		2007	0.11
		2008	0.11
		2009	0.09
		2010	0.08
		2011	0.10

ABC, Active Bacterial Core Surveillance; EMDS, Enhanced Meningococcal Disease Surveillance; MenY, meningococcal serogroup Y.

a Includes California (3-county San Francisco Bay area), Colorado (5-county Denver area), Connecticut, Georgia, Maryland, Minnesota, New Mexico, New York (15-county Rochester and Albany areas), and Tennessee (20 counties).

### MenY in Africa

In South Africa, the number of MenY cases across all ages decreased from 44 in 2010 to 27 in 2019 [24, 25]. A 2012 article also documented a decrease in the incidence rates of MenY IMD, declining substantially from 0.11/100,000 in 2005 to 0.04/100,000 in 2008 in all ages [13]. Recently, five sub-Saharan countries (Burkina Faso, Niger, Chad, Mali, and Togo) reported an average incidence rate of 0.01/100,000 during 2015–2017 (Table 1) [14]. In 13 sub-Saharan countries (Benin, Burkina Faso, Cameroon, Central African Republic, Chad, Ghana, Kenya, Mali, Niger, Nigeria, Sudan, Togo, and Uganda), 3% of the IMD cases were due to MenY between 2004 and 2010 in all ages [26]. A comparatively higher proportion of MenY IMD was reported in South Africa from 2003 to 2016 in all ages (10%–12.3%) [13, 27].

### MenY IMD in Asia

In general, trends in Asia varied by region and period studied, with some reporting stable MenY IMD. No incidences of MenY disease were reported between 2000 and 2002 in Vietnam [28]. A relatively high proportion of MenY-attributed disease was observed in Japan, accounting for 42% of IMD between 2013 and 2014 in all ages [29]. Similar proportions of MenY were observed in South Korea (38%) during 1999–2001 and China (33%) during 2000–2002 in young children <5 years of age [28]. In Turkey, an increase in MenY disease was observed, rising from 1% to 3% in individuals <18 years of age between 2005–2012 and 2013–2014, respectively [30, 31]. Israel also observed an increase, with MenY IMD rising from 11% during 2007–2013 to 20% during 2014–2017 in all ages [32].

### MenY IMD in Oceania

The incidence of MenY disease in Australia fluctuated between 0.02 and 0.07 per 100,000 population between 1999 and 2014 and then increased to 0.09 per 100,000 population in 2015 (Table 1) [15]. The number of MenY IMD cases has increased from 2012 onwards, peaking in 2017, and has recently begun to trend downwards thereafter (Figure 2 and Supplementary Figure S2). Similarly, the proportion of IMD cases accounted for by MenY increased from 2010 to 2019 [12]. Similar increases have been observed in the state of Victoria, with MenY disease rising from 9% during 2008–2012 to 16.8% from July 2015 to December 2017 in all ages [33, 34]. The number of MenY cases in each age group generally increased between 2012 and 2017 and then decreased between 2017 and 2019 (Figure 2) [12]. Over time, the greatest proportions of cases were accounted for by adolescents and young adults aged 15–24 years, adults aged 45–64 years, and adults ≥65 years of age. Overall, MenY trends in New Zealand were similar to those in Australia, with MenY representing only 3%–7% during 2010–2013, reaching 15% and 13% in 2018 and 2019, respectively [35]. The total number of MenY cases over time compared with selected regions is shown in Supplementary Figure S2.

### MenY IMD in Europe

Based on data from the ECDC, the incidence of MenY in all ages fluctuated from 0.00 to 0.72 in the European Union/European Economic Area region during 2010–2018 (Supplementary Table S1), with MenY accounting for 6%–12% of overall cases of IMD (Figure 3a) [10]. Age-specific incidence rates varied substantially, specifically fluctuating in children <1 year of age and showing a gradual increase in adults >65 years (Figure 3b). Estonia, Lithuania, and Luxembourg reported no incidence of MenY disease during this period. In 2018, the highest incidence rates were observed in Belgium (0.25/100,000) and Norway (0.23/100,000). Overall and age-specific trends in incidence rate (Supplementary Figure S1 and Table S1) and number/proportion of MenY among all IMD (Supplementary Figure S3 and Table S2) varied by country. Trends showing increasing incidence of MenY through 2017–2018 were observed in Belgium, Germany, Italy, the Netherlands, and Spain. Rates in Finland and Norway have fluctuated from 2010 to 2018, not showing a specific trend over time, whereas rates have been decreasing in Sweden since 2013.

### MenY IMD in Latin America

**Figure 4.**
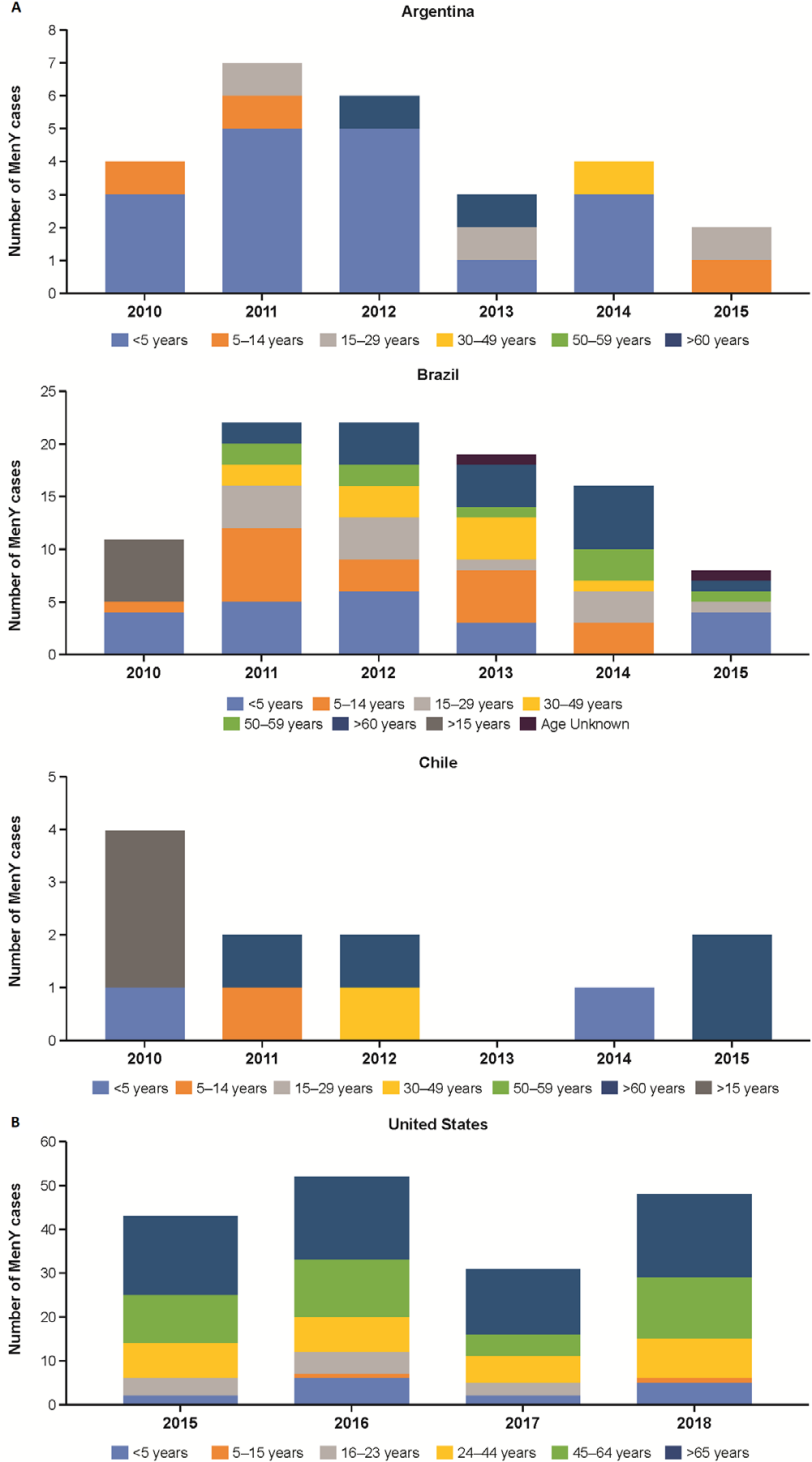
Number of MenY IMD cases by age group in (a) selected countries in Latin America [36] and (b) the United States [19]. IMD, invasive meningococcal disease; MenY, meningococcal serogroup Y.

The percentage of MenY IMD was high in Latin America during the study period, accounting for up to 100% of grouped IMD case isolates in El Salvador (2012), Honduras (2012), and Costa Rica (2010) among all age groups [36]. However, cases of MenY IMD are trending downwards in some Latin American countries (Figure 4a); for example, in Argentina, the number of IMD cases accounted for by MenY peaked in 2011 (n = 7) and then generally decreased through 2015 (n = 2; Figure 4a) [36]. By contrast, incidence rates in Brazil ranged from 0.03/100,000 between 2005 and 2009 to 0.04/100,000 in 2011 (Table 1) [16], with the proportion of IMD cases accounted for by MenY in Brazil increasing from 1.7% in 2010 to 5.3% in 2014 among all ages [36]. Similar to Argentina, the highest number of cases in Brazil occurred in 2011 and 2012 (n = 22 each) and then declined through 2015 (n = 7; Figure 4a) [36]. A similar trend was observed in Chile, where the number of MenY cases was highest in 2010 (n = 4) and then generally decreased through 2015 (n = 2; Figure 4a) [36].

### MenY IMD in North America

Incidence rates of MenY disease in North America have declined over the past two decades, but absolute case numbers and incidence rates remain high, particularly among adults ≥65 years of age (Figure 4b and Tables 1 and 2). The percentage of MenY disease in the United States observed between 2006 and 2015 ranged from 7.0% to 43.6% depending on the age group [6]; across all ages, the percentage of MenY IMD decreased from 33.4% during 2000–2005 to 27.3% during 2011–2015 [38]. The incidence of MenY disease in the United States between 2006 and 2015 ranged from 0.02/100,000 to 0.35/100,000 depending on age group (Table 2) [6]. According to the Enhanced Meningococcal Disease Surveillance system, incidence of MenY disease has generally remained stable in recent years (Figure 4b) [19]. Overall, Canada appears to have stable disease, with a reported 18.0% of MenY disease between 2006 and 2011 overall and incidence rates evolving from 0.08/100,000 in 2006 and 0.10/100,000 in 2011 (Table 2) [21]. In British Columbia and Quebec, the overall (not stratified by age) number of MenY cases increased from 2015 to 2017 [39–41].

**Table 2.** Incidence of MenY disease by age group in North America

Publication	Country	Timeperiod	Age group, y	Incidence MenY (per 100,000)
*North America*				
Cohn et al. [17]	United States	1998–2007	<1	1.5
			1	0.31
			2–4	0.12
			5–9	0.06
			10–13	0.09
			14–17	0.29
			18–24	0.15
			25–64	0.11
			≥65	0.42
MacNeil et al. [6]	United States	2006–2015	<1	0.35
			1	0.08
			2–4	0.02
			5–10	0.02
			11–15	0.03
			16–20	0.08
			21–25	0.05
			26–44	0.03
			45–64	0.05
			65–84	0.12
			≥85	0.26
Wormsbecker et al. [37]	Canada (Ontario)	2000–2009	<1	0.37
			1–4	0.09
			5–11	0.06
			12–16	0.14
			17–21	0.21
			22–29	0.07
			30–44	0.04
			45–64	0.10
			≥65	0.25
		2010–2013	<1	0.36
			1–4	0.09
			5–11	0
			12–16	0.09
			17–21	0.14
			22–29	0.05
			30–44	0.05
			45–64	0.09
			≥65	0.15

MenY, meningococcal serogroup Y.

### MenY and different clinical manifestations

Clinical manifestations of MenY cases were not necessarily and specifically noted in the reports and publications. Only the ECDC reports were extractable for MenY specific clinical manifestations data. For all ages from 2010 to 2019, most of reported MenY cases, 63% (46% in 2018 to 74% in 2012), did not indicate their clinical manifestations, while 12% were meningitis, 15% septicemia, and 6% meningitis + septicemia. Pneumonia cases were mostly reported starting from 2015 and almost exclusively occurred in patients 50 years and older despite its small proportion (1%) among all MenY cases. Among all reported MenY cases in those 50 years and older in 2019, septicemia represented 27% of cases, meningitis 9% of cases, and unknown cases remained high at 50%.

## Discussion

The changing epidemiology of meningococcal disease continues to present challenges owing to significant differences in serogroup distribution depending on the geographic region [4]. MenY disease is highly variable throughout the world, affecting countries differently over time. This review highlights the evolution of MenY disease over the past two decades and demonstrates the need for continued surveillance efforts of MenY disease moving forward. Much of the data presented in this review were based on surveillance reports rather than publications, as these provided more current and/or granular information.

The incidence of MenY was relatively low compared to other serogroups, but significantly increased in some regions during the last decade [10]. In Europe during 2008–2017, decreasing trends in all age groups were observed for serogroups B and C, while increasing trends were observed for serogroups W and Y (MenY, 137% increase; MenW, 517% increase) [11]. Rates of MenY varied considerably by location, age group, and over time. Notably, MenY cases were increasing in Belgium, Germany, Italy, the Netherlands, and Spain prior to MenACWY vaccine introduction in some of the countries [10, 23]. Similar trends were observed in Australia [12] and Israel [32].

Epidemiology of MenY varied by age, with the burden of disease generally higher in older adults (≥65 years of age; Supplementary Figure S3). However, the incidence of MenY in older adults could be underestimated on account of frequent non-meningitis clinical presentations [42]. It is also noteworthy that MenY was not part of the specific reporting serogroups in many countries and microbiological testing for serogroups may be less frequent for respiratory cases than meningitis cases. Recent studies, however, described an independent association between increasing age and bacteremic meningococcal pneumonia in England [43], and an increase in respiratory forms of IMD among elderly individuals in Netherlands and France [44]. The recent increase in MenY cases could be due to increasing awareness of non-meningitis clinical presentations specifically in older adults or could be a true increase in incidence rates. It is thus important to interpret trends with precaution and closely follow-up the MenY respiratory forms specifically in the context of post-COVID periods.

The epidemiology of MenY varied among countries and incidence rates generally decreased in regions where MenACWY immunization programs were implemented [10, 23]. Notably, the use of MenACWY vaccines in several countries in recent years has shown substantial benefits, particularly in the United States, with the incidence of MenY disease decreasing overall in recent years (for ages <20 years, incidence of serogroups A, C, W, and Y declined 20%–95% between 2008 and 2015) [6, 22]. Other countries such as the Netherlands, Australia, and England have also observed a similar benefit, with MenY cases decreasing in recent years since implementing MenACWY vaccination programs [10, 23]. Such decreases were observed not only within vaccine-eligible age groups (i.e., young children and adolescents), but also within other age groups and particularly among older adults, for whom high case numbers had been observed.

To our knowledge, this is the first review to provide a comprehensive overview of the burden of MenY IMD globally. However, there are some limitations to our review, including the lack of available literature and contemporaneous data sets compared with other meningococcal serogroups (MenB, MenC, and MenW). The number of cases and incidence of disease are also considerably lower compared with other serogroups; this presented challenges when interpreting the data, especially as it pertained to accounting for fluctuations over time that could impact trend analyses. Also, for the report on the 13 sub-Saharan countries, more than one-third of all samples collected were from Burkina Faso, with approximately one-third from Mali and Niger combined [26]. Moreover, interpretation of changes in proportion of MenY may have been different between percentage changes and changes in case numbers (e.g., although the same number of MenY cases were observed, the percentage of MenY increased due to fewer cases being observed with another serogroup). The changes in proportions described throughout the review should be interpreted along with the total number of cases.

Overall, our study highlights the variability of MenY IMD from region to region and demonstrates the increased burden of disease in individuals ≥65 years of age. These data can change over time due to changes in the population size and composition, meningococcal vaccination programs, and secular trends [45]. However, they can be used to inform disease burden estimates and future vaccination policies. Continuous monitoring of the epidemiologic changes of meningococcal disease is essential to protection and prevention efforts. In this analysis, we report data from selected countries to make key points. Careful comparison with other data presented should be done to ensure consistency with other countries for which data were not included in this analysis. To that end, countries should continue to employ surveillance strategies to capture true estimates of the burden of MenY disease worldwide.

## Supporting information

Tin Tin Htar et al. supplementary material Tin Tin Htar et al. supplementary material

## Data Availability

This article is based on published literature and therefore does not contain any applicable data sets.

## Supplementary material

For supplementary material accompanying this paper visit http://doi.org/10.1017/S0950268824001535. click here to view supplementary material
