# Supplementary material for: Global epidemiology of serogroup Y invasive meningococcal disease: a literature review
Source: Epidemiol Infect. 2024 Dec 5;152:e157. doi: 10.1017/S0950268824001535 (PMC11648504; doi:10.1017/S0950268824001535)
Supplement: Tin Tin Htar et al. supplementary material [file S0950268824001535sup001.docx]

# ***Epidemiology and Infection***

**Global Epidemiology of Serogroup Y Invasive Meningococcal Disease: A Literature Review**

Myint Tin Tin Htar, Jamie Findlow, Paul Balmer, and David Swerdlow

# **Supplementary Material**

**Supplementary Text: Search String**

**Supplementary Table S1: Incidence of MenY in European countries in all ages**

**Supplementary Table S2. Proportion of MenY among all IMD in all ages in the EU/EEA**

**Supplementary Figure S1. Incidence of MenY by age group in select European countries**

**Supplementary Figure S2. Total Number of MenY among all IMD in select regions**

**Supplementary Figure S3. Number of MenY among all IMD by age group in select European countries**

**Supplementary Text**

*Search String*

Search string: ((((((((((serogroup) OR serotype) OR strain)) AND (((meningo*) OR meningitidis) OR meningitides))) NOT (((((cost*[Title]) OR econo*[Title]) OR model*[Title])) OR ((immunogenicity[Title]) OR bactericidal[Title])))) NOT ((("adaptive clinical trial"[Publication Type] OR "case reports"[Publication Type] OR "clinical trial"[Publication Type] OR "clinical trial protocol"[Publication Type] OR "clinical trial, phase I"[Publication Type] OR "clinical trial, phase II"[Publication Type] OR "clinical trial, phase III"[Publication Type] OR "clinical trial, phase iv"[Publication Type] OR "controlled clinical trial"[Publication Type] OR "editorial"[Publication Type] OR "guideline"[Publication Type] OR "randomized controlled trial"[Publication Type] OR "review"[Publication Type] OR "validation studies"[Publication Type]))))) AND ("2010/01/01"[Date - Publication] : "2021/25/03"[Date - Publication])

|  | **2010** | **2011** | **2012** | **2013** | **2014** | **2015** | **2016** | **2017** | **2018** |
| --- | --- | --- | --- | --- | --- | --- | --- | --- | --- |
| EU/EEA | 0.04 | 0.06 | 0.05 | 0.06 | 0.05 | 0.06 | 0.07 | 0.07 | 0.07 |
| Austria | 0.05 | 0.02 | 0.02 | 0.05 | 0.06 | 0.02 | 0.02 | 0.02 | 0.02 |
| Belgium | 0.04 | 0.08 | 0.08 | 0.06 | 0.11 | - | 0.15 | 0.17 | 0.25 |
| Bulgaria | - | - | - | - | - | - | - | - | - |
| Croatia | - | - | - | - | - | - | - | - | - |
| Cyprus | 0.00 | 0.00 | 0.12 | 0.00 | 0.00 | 0.00 | 0.00 | 0.00 | - |
| Czechia | 0.04 | 0.02 | 0.01 | 0.02 | 0.01 | 0.01 | 0.01 | 0.01 | 0.03 |
| Denmark | 0.02 | 0.09 | 0.11 | 0.07 | 0.09 | 0.07 | 0.05 | 0.14 | 0.03 |
| Estonia | 0.00 | 0.00 | 0.00 | - | - | - | 0.00 | 0.00 | 0.00 |
| Finland | 0.24 | 0.13 | 0.15 | 0.15 | 0.09 | 0.05 | 0.09 | 0.16 | 0.09 |
| France | 0.04 | 0.07 | 0.05 | 0.09 | 0.06 | 0.08 | 0.09 | 0.12 | 0.09 |
| Germany | 0.02 | 0.02 | 0.02 | 0.03 | 0.02 | 0.03 | 0.04 | 0.04 | 0.04 |
| Greece | 0.00 | 0.00 | 0.03 | 0.01 | 0.02 | 0.04 | 0.02 | 0.02 | 0.02 |
| Hungary | 0.00 | 0.00 | 0.00 | 0.03 | 0.01 | 0.00 | 0.00 | 0.01 | 0.00 |
| Iceland | 0.00 | 0.00 | 0.00 | 0.00 | 0.00 | 0.00 | 0.00 | 0.30 | 0.00 |
| Ireland | 0.00 | 0.02 | 0.04 | 0.04 | 0.04 | 0.15 | 0.11 | 0.02 | 0.17 |
| Italy | 0.02 | 0.03 | 0.03 | 0.03 | 0.02 | 0.04 | 0.04 | 0.06 | 0.05 |
| Latvia | 0.00 | 0.00 | 0.00 | 0.00 | 0.00 | 0.05 | 0.00 | 0.00 | 0.00 |
| Lithuania | - | 0.00 | 0.00 | 0.00 | 0.00 | 0.00 | 0.00 | 0.00 | 0.00 |
| Luxembourg | 0.00 | - | - | - | 0.00 | - | 0.00 | 0.00 | 0.00 |
| Malta | 0.00 | 0.72 | 0.00 | 0.47 | 0.00 | 0.00 | 0.22 | 0.00 | 0.21 |
| Netherlands | 0.06 | 0.08 | 0.09 | 0.06 | 0.07 | 0.04 | 0.10 | 0.16 | 0.14 |
| Norway | 0.27 | 0.41 | 0.12 | 0.14 | 0.10 | 0.15 | 0.17 | 0.15 | 0.23 |
| Poland | 0.01 | 0.01 | 0.01 | 0.00 | 0.01 | 0.02 | 0.01 | 0.01 | 0.01 |
| Portugal | 0.00 | 0.09 | 0.04 | 0.03 | 0.04 | 0.07 | 0.04 | 0.04 | 0.05 |
| Romania | - | 0.00 | - | 0.00 | 0.01 | 0.00 | 0.01 | - | - |
| Slovakia | 0.00 | 0.02 | 0.00 | 0.00 | - | 0.00 | 0.02 | 0.00 | 0.00 |
| Slovenia | 0.05 | 0.10 | 0.00 | 0.00 | 0.05 | 0.00 | 0.10 | 0.00 | 0.10 |
| Spain | 0.00 | 0.01 | 0.01 | 0.01 | 0.01 | 0.01 | 0.04 | 0.04 | 0.10 |
| Sweden | 0.25 | 0.34 | 0.47 | 0.41 | 0.18 | 0.17 | 0.18 | 0.14 | 0.15 |
| United Kingdom | 0.11 | 0.15 | 0.14 | 0.14 | 0.14 | 0.19 | 0.18 | 0.12 | 0.12 |

# **Supplementary Table S1: Incidence of MenY in European countries in all ages [10]**

EEA=European Economic Area; EU=European Union; MenY=meningococcal serogroup Y.

# **Supplementary Table S2. Proportion of MenY among all IMD in all ages in the EU/EEA [10]**

|  | **2010** | **2011** | **2012** | **2013** | **2014** | **2015** | **2016** | **2017** | **2018** |
| --- | --- | --- | --- | --- | --- | --- | --- | --- | --- |
| Austria | 4.9 | 7.1 | 4.8 | 7.1 | 14.3 | 9.1 | 6.5 | 10.5 | 8.3 |
| Belgium | 4.4 | 8.2 | 7.3 | 5.2 | 14.0 | - | 16.0 | 19.8 | 25.0 |
| Bulgaria | - | - | - | - | - | - | - | - | - |
| Croatia | - | - | - | - | - | - | - | - | - |
| Cyprus | 0.0 | - | 16.7 | 0.0 | 0.0 | 0.0 | 0.0 | 0.0 | - |
| Czechia | 8.2 | 5.0 | 1.8 | 3.8 | 2.7 | 2.2 | 2.6 | 1.6 | 5.6 |
| Denmark | 1.6 | 6.9 | 10.7 | 10.0 | 14.3 | 18.2 | 9.1 | 23.5 | 5.4 |
| Estonia | 0.0 | 0.0 | 0.0 | - | - | - | 0.0 | 0.0 | 0.0 |
| Finland | 40.6 | 21.2 | 26.7 | 40.0 | 27.8 | 15.0 | 27.8 | 56.3 | 31.3 |
| France | 5.5 | 8.3 | 5.6 | 10.0 | 9.7 | 11.9 | 12.3 | 14.6 | 13.3 |
| Germany | 5.4 | 5.6 | 4.1 | 7.8 | 7.2 | 8.0 | 10.9 | 14.1 | 14.0 |
| Greece | 0.0 | 0.0 | 6.1 | 2.0 | 3.6 | 8.0 | 4.5 | 6.3 | 6.1 |
| Hungary | 0.0 | 0.0 | 0.0 | 6.7 | 3.4 | 0.0 | 0.0 | 2.6 | 0.0 |
| Iceland | 0.0 | 0.0 | 0.0 | 0.0 | 0.0 | 0.0 | - | 33.3 | - |
| Ireland | 0.0 | 1.1 | 3.3 | 2.7 | 2.6 | 10.3 | 6.0 | 1.4 | 9.1 |
| Italy | 10.0 | 13.6 | 16.7 | 15.7 | 13.9 | 16.1 | 12.9 | 18.7 | 17.6 |
| Latvia | 0.0 | 0.0 | 0.0 | 0.0 | 0.0 | 12.5 | 0.0 | 0.0 | 0.0 |
| Lithuania | - | 0.0 | 0.0 | 0.0 | 0.0 | 0.0 | 0.0 | 0.0 | 0.0 |
| Luxembourg | 0.0 | - | - | - | 0.0 | - | 0.0 | - | 0.0 |
| Malta | 0.0 | 50.0 | 0.0 | 22.2 | 0.0 | 0.0 | 25.0 | 0.0 | 25.0 |
| Netherlands | 7.7 | 13.1 | 15.3 | 9.9 | 15.8 | 7.9 | 11.2 | 13.6 | 11.7 |
| Norway | 33.3 | 54.1 | 25.0 | 26.9 | 29.4 | 42.1 | 37.5 | 44.4 | 46.2 |
| Poland | 2.4 | 0.8 | 0.9 | 0.5 | 2.3 | 3.3 | 1.3 | 1.0 | 1.6 |
| Portugal | 0.0 | 14.9 | 7.3 | 5.6 | 9.8 | 11.7 | 11.4 | 9.8 | 8.9 |
| Romania | - | 0.0 | - | 0.0 | 2.5 | 0.0 | 5.1 | - | - |
| Slovakia | 0.0 | 6.7 | 0.0 | 0.0 | - | 0.0 | 4.8 | 0.0 | 0.0 |
| Slovenia | 11.1 | 15.4 | 0.0 | 0.0 | 12.5 | 0.0 | 33.3 | 0.0 | 11.1 |
| Spain | 0.5 | 1.0 | 1.6 | 2.4 | 3.0 | 3.0 | 7.9 | 7.6 | 13.1 |
| Sweden | 34.3 | 51.6 | 45.5 | 54.2 | 37.8 | 35.4 | 32.1 | 31.1 | 29.4 |
| United Kingdom | 7.2 | 9.6 | 10.4 | 10.9 | 11.7 | 13.2 | 13.5 | 10.6 | 10.5 |

EEA=European Economic Area; EU=European Union; IMD=invasive meningococcal disease; MenY=meningococcal serogroup Y.

# **Supplementary Figure S1. Incidence of MenY by age group in select European countries [10].**


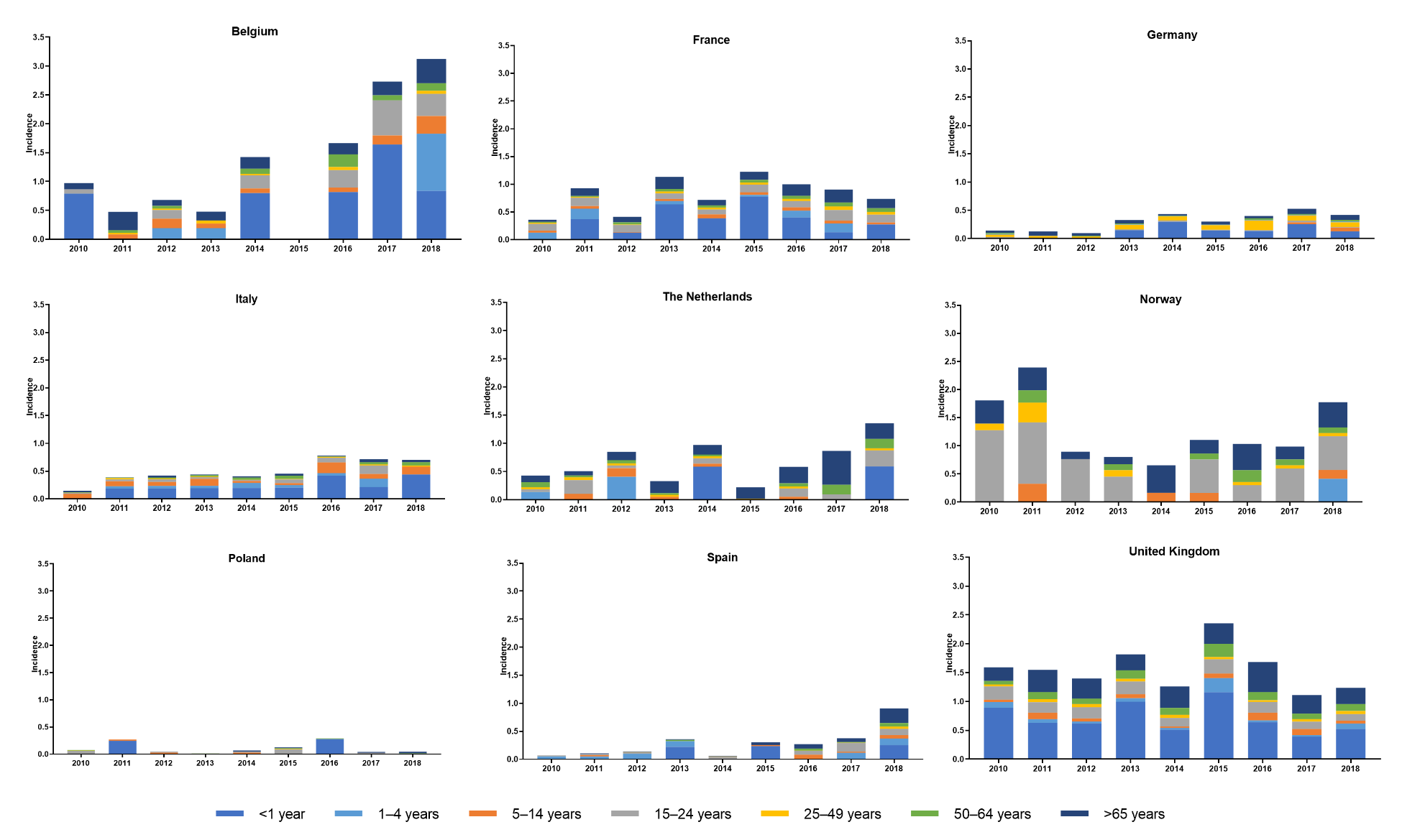


EEA=European Economic Area; EU=European Union; IMD=invasive meningococcal disease; MenY=meningococcal serogroup Y.

# **Supplementary Figure S2. Total Number of MenY among all IMD in select regions [10,26,30,33].**

MenY=meningococcal serogroup Y.

# **Supplementary Figure S3. Number of MenY among all IMD by age group in select European countries^a^ [10].**

IMD=invasive meningococcal disease; MenY=meningococcal serogroup Y.

^a^Figure panels are not all on the same Y-axis scale to ensure better visibility of data across countries with low MenY cases.
